# Hybrid magnetoresistance in Pt-based multilayers: Effect originated from strong interfacial spin-orbit coupling

**DOI:** 10.1038/srep20522

**Published:** 2016-02-04

**Authors:** Kangkang Meng, Jiaxing Xiao, Yong Wu, Jun Miao, Xiaoguang Xu, Jianhua Zhao, Yong Jiang

**Affiliations:** 1 School of Materials Science and Engineering, University of Science and Technology Beijing, Beijing 100083, China; 2 State Key Laboratory of Superlattices and Microstructures, Institute of Semiconductors, Chinese Academy of Sciences, Beijing 100083, China

## Abstract

The hybrid magnetoresistance (MR) behaviors in Pt/Co_90_Fe_10_/Pt, Mn_1.5_Ga/Pt and Mn_1.5_Ga/Pt/Co_90_Fe_10_/Pt multilayers have been investigated. Both planer Hall effect (PHE) and angle-dependent MR in Pt/Co_90_Fe_10_/Pt revealed the combination of spin Hall MR (SMR) and normal anisotropic MR (AMR), indicating the large contribution of strong spin-orbit coupling (SOC) at the interfaces. When Pt contacted with perpendicular magnetic anisotropy (PMA) metal Mn_1.5_Ga, the strong interfacial SOC modified the effective anomalous Hall effect. The MR in Mn_1.5_Ga/Pt/Co_90_Fe_10_/Pt is not a simple combination of SMR and AMR, but ascribed to the complicated domain wall scattering and strong interfacial SOC when Pt is sandwiched by the in-plane magnetized Co_90_Fe_10_ and the PMA Mn_1.5_Ga.

Magnetoresistance (MR) is the property of a material to change the value of its electrical resistance under an external magnetic field. The dependence of resistance on the angle between current and magnetization in metallic ferromagnets (FM) is called anisotropic magnetoresistance (AMR)^1^. On the other hand, planar Hall effect (PHE) and anomalous Hall effect (AHE) are both observed as a voltage transverse to the applied current in contrast to AMR, which is measured in longitudinal geometry^2^,^3^,^4^,^5^. The longitudinal resistivity ρ_xx_ denoting AMR and the transverse resistance ρ_xy_ characterizing PHE are given by:


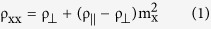



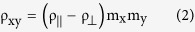


where ρ_‖_ (ρ_⊥_) is the resistivity along (perpendicular to) the direction of the in-plane component of magnetization (**M)**, m_x_, m_y_ are the components of the in-plane magnetization along *x* and *y* directions respectively. For the ordinary AMR effect in magnetic layers, ρ_xx_ only depends on the magnetization component parallel to current.

Recently, a new type of MR is observed when a strong spin-orbit coupling (SOC) metal such as Pt comes in contact with a FM, either metallic or insulating^6^,^7^,^8^,^9^,^10^,^11^,^12^,^13^,^14^,^15^. In these hybrid structures, spin and charge transport phenomena are interconnected, and Pt may serve as both spin current generator and detector^12^,^13^,^14^,^15^,^16^. The spin Hall effect (SHE) can convert charge current into pure spin current in the transverse direction and the conversion is enhanced in heavy metals such as Pt due to their strong SOC. The spin current can be used to apply torque to magnetic moment by direct transfer of spin angular momentum^17^,^18^,^19^,^20^. On the other hand, it can also be detected by inverse spin Hall effect (ISHE), which converts the pure spin current into charge current resulting in charge accumulation along the transverse direction. Nakayama *et al*. had presented the unusual MR of Pt/ yttrium iron garnet (YIG) in terms of a nonequilibrium proximity effect caused by the simultaneous action of SHE and ISHE and therefore called it spin Hall MR (SMR)^12^. The experiments were theoretically explained by Chen *et al*. who proposed a SMR theory based on the spin-diffusion approximation in a Pt layer in the presence of spin-orbit interaction and quantum mechanical boundary conditions at the Pt/YIG interface in terms of spin-mixing conductance^13^. At the interface the electrons in Pt will interact with the localized moment in the FM. A part of spin current is absorbed by the magnetization as spin-transfer torque and the spin-current reflection is thus suppressed. This absorption is zero when the magnetization **M** is parallel to the spin-current polarization **σ** and maximized when **M** is perpendicular to **σ**. By changing the magnetization direction of the FM, the polarization direction of the reflected spins and thus the direction of the additional created charge current can be controlled, and a transverse voltage is also generated. In a word, the SMR is a strong interfacial SOC phenomenon.

However, the reports of SMR, so far, have mostly focused on Pt/YIG bilayers, because one can easily access the magnetotransport properties of the Pt thin film deposited on the insulating FM YIG. There is a challenge to detect the mechanism of the strong interfacial SOC when Pt contacts with a normal FM such as CoFe. Meanwhile, how about the phenomena when Pt contacts with perpendicular magnetic anisotropy (PMA) metals since SMR is also influenced by perpendicular magnetization component? In the past two decades, PMA Mn_*x*_Ga (1 < *x* < 1.8) alloy thin films with L1_0_ structure have gained increasing attention for possible application in ultrahigh density magnetic recording media, permanent magnets and spintronics^21^,^22^. Therefore, there is also a fundamental interest to explore the spin current related phenomena when Pt contacts with a PMA Mn_*x*_Ga.

In this work, we have investigated MR behaviors in Pt/Co_90_Fe_10_/Pt, Mn_1.5_Ga/Pt and Mn_1.5_Ga/Pt/Co_90_Fe_10_/Pt multilayers (Co_90_Fe_10_ and Mn_1.5_Ga will be simply noted as CoFe and MnGa in the following paragraph), in which CoFe is polycrystalline and MnGa is a single-crystalline PMA metal. The magnetic and transport properties are compared with the multilayers in which Pt is replaced by Cu with a weak SOC. The PHE of Pt/CoFe/Pt is much larger than that of Cu/CoFe/Cu. On the other hand, as compared with normal AMR in Cu/CoFe/Cu, the angle-dependent MR in Pt/CoFe/Pt reveals that the longitudinal resistivity change is also related with the magnetization perpendicular to the current direction in the film plane. The phenomenon indicates a large contribution of strong SOC at the interface. When Pt contacts with PMA MnGa, the effective AHE becomes smaller, which also confirms the strong interfacial scattering due to SOC. The MR in MnGa/Pt/CoFe/Pt is not a simple combination of SMR and AMR but ascribed to the complicated domain wall scattering and SOC when Pt is sandwiched by the in-plane CoFe and the PMA MnGa.

## Results

### Magnetic properties

**Figure 1:**
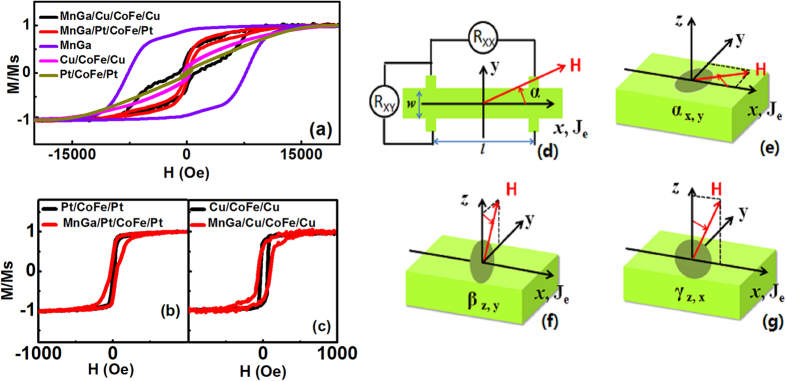
Magnetic properties. (**a–c**) The normalized out-of-plane and in-plane magnetic hysteresis loops at 300 K. (**d**) Schematic diagram of the longitudinal (R_XX_) and transverse (R_XY_) resistance measurements. The charge current **J**_**e**_ is along ***x*** axis. (**e–g**) Schematic diagram of the magnetic field applied in ***xy, zy,*** and ***zx*** planes with angles α_xy_, β_zy_ and γ_zx_, respectively.

The out-of-plane and in-plane hysteresis loops of all the fabricated multilayers and a single 20-nm-thick MnGa film are shown in Fig. 1a–c. The perpendicular **M-H** curve of MnGa shows a square-like shape revealing its giant PMA. Although Pt/CoFe/Pt and Cu/CoFe/Cu exhibit similar magnetization reversal in perpendicular component, the M-H curves of MnGa/Pt/CoFe/Pt and MnGa/Cu/CoFe/Cu show different domain structures. Both Pt/CoFe/Pt and Cu/CoFe/Cu show typical in-plane magnetic anisotropy and have almost the same saturated magnetization. However their coercivities are different, indicating delicately different micromagnetic configurations of domain walls. Both coercivities and saturation fields of MnGa/Cu/CoFe/Cu and MnGa/Pt/CoFe/Pt have been enhanced and their values are almost the same as shown in the curves parallel to the plane in Fig. 1b,c.

All the samples were fabricated into Hall bars with a nominal length *l* of 2.5 mm and a width *w* of 0.2 mm. Figure 1d shows the resistance measurement geometry of the Hall bars in the ***xy*** plane with a current along ***x*** and the configurations for longitudinal R_XX_ and transverse resistance R_XY_. For subsequent measurements, the magnetic field was applied in the ***xy, zy,*** and ***zx*** planes with angles α_xy_, β_zy_ and γ_zx_ (simply noted as α, β and γ) respectively, as shown in Fig. 1e–g.

### Hybrid MR in Pt/CoFe/Pt

**Figure 2:**
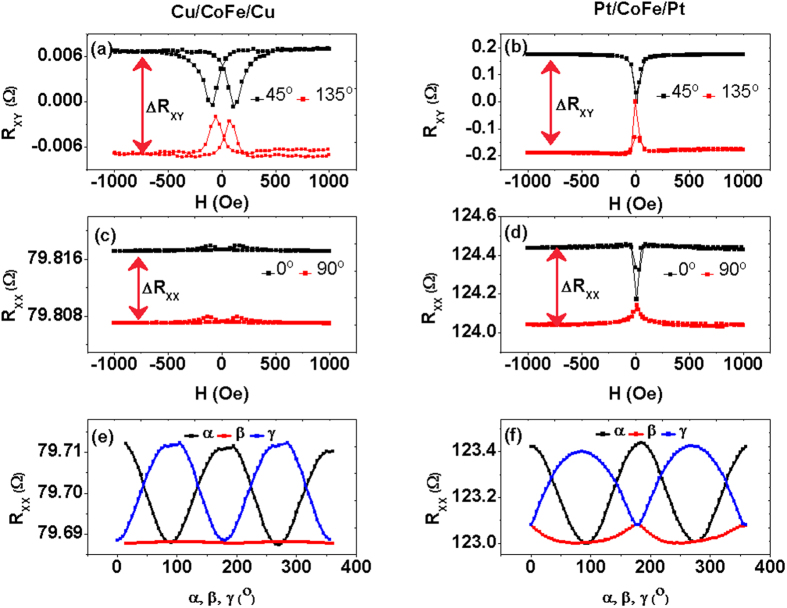
PHE and MR of Cu/CoFe/Cu and Pt/CoFe/Pt. (**a,b**) Measurements of R_XY_ in Cu/CoFe/Cu and Pt/CoFe/Pt with the applied magnetic field forming a fixed angle (α = 45° and α = 135°) with the current. (**c,d**) Measurements of R_XX_ with the applied magnetic field forming a fixed angle (α = 0° and α = 90°) with the current. (**e,f**) The α, β and γ dependence of R_XX_ with a magnetic field of 6 T.

The AMR (R_XX_) and PHE (R_XY_) measured at low magnetic field (**H** = 1000 Oe) and the angle-dependent MR measured at high field (**H** = 6 T) of Pt/CoFe/Pt and Cu/CoFe/Cu are shown in Fig. 2. In Fig. 2a,b, the measurements of PHE were done with the applied magnetic field forming a fixed angle (α = 45° and 135°) with the current, since the signal will be maximized in this geometry as shown in equation (2). After subtracting the common offset, the signals with opposite sign were obtained. On the other hand, the AMR measurements with maximized signals were done with the applied field keeping a fixed angle with the current (α = 0° and 90°) shown in Fig. 2c,d. It is observed that both the resistance change ΔR_XX_ and ΔR_XY_ of Pt/CoFe/Pt are all much larger than those of Cu/CoFe/Cu. Considering the thickness and polycrystalline structure of the CoFe layer, the MR caused by magnetic domain walls for the two multilayers should all be quite small. The enhancement of the resistance change in Pt/CoFe/Pt may mostly be contributed by SMR. The longitudinal and transverse resistivity change for SMR can be formulated as^13^:


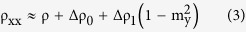



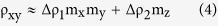


where ρ is the intrinsic electric resistivity, Δρ_0_ is the resistivity reduced by the spin-orbit interaction, m_z_ is the component of the magnetization in ***z*** direction. Δρ_1_ and Δρ_2_ are the magnitude of the resistivity related to the complex spin-mixing interface conductance G_↑↓_ = G_r_ + iG_i_. Δρ_1_ (caused mainly by G_r_) contributes to the conductance modulation depending on the in-plane component of the magnetization, while Δρ_2_ (caused mainly by G_i_) contributes only when there is a magnetization component normal to the plane. Therefore, the resistance change not only depends on m_x_ in ordinary AMR but also on m_y_ in SMR. Meanwhile, for both longitudinal and transverse configurations, there are peaks or dips observed around the coercivity, and they also depend on the field direction. It is proposed that the magnetization of CoFe will be fully rotated in-plane towards **H** due to its in-plane magnetic anisotropy. This magnetic rotation results in a change in measured resistance, passing the maximum or minimum resistance, which is observed as a peak or dip around the coercivity. Figure 2e,f show the α, β and γ dependence of R_XX_ in Pt/CoFe/Pt and Cu/CoFe/Cu respectively. Cu/CoFe/Cu exhibits a normal AMR of a polycrystalline ferromagnetic film with in-plane anisotropy: R_XXL_ > R_XXT_, R_XXT_ ≈ R_XX⊥_^1^, where R_XXL_, R_XXT_ and R_XX⊥_ are longitudinal resistances when α = 0^o^, α = 90^o^ and β = 0^o^ respectively. For Pt/CoFe/Pt, 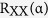 shows the cos^2^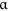 dependence but have a feature of R_XXL_ > R_XX⊥_ > R_XXT_, which is different from pure AMR or SMR because the 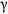 scan of 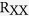 does not keep constant^12^. R_XX_(β) shows the −sin^2^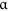 dependence, which is consistent with equation (3). Furthermore, the SMR (Δρ_1_/ρ) can be formulated as^13^


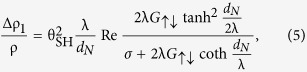


where θ_SH_ is the spin hall angle, *d*_*N*_ the thickness of heavy metal layer, σ = ρ^−1^ the conductivity and λ the spin diffusion length. By fitting the angular dependence curves in Fig. 2f, we firstly obtain Δρ_1_ = 6.4 × 10^−3^ μΩcm and SMR = Δρ_1_/ρ ≈ 0.06%. Using the parameters θ_SH_ = 0.05 and λ = 1.5 nm for Pt^13^, the spin mixing conductance G_↑↓_ of the multilayers can be deduced from Eq. (5) as about 2.6 × 10^10^ Ω^−1^m^−2^. All the results reveal the combination of SMR and normal AMR, indicating the large contribution of strong SOC at the interfaces.

### Spin current related transport properties of the PMA MnGa/Pt bilayers

**Figure 3:**
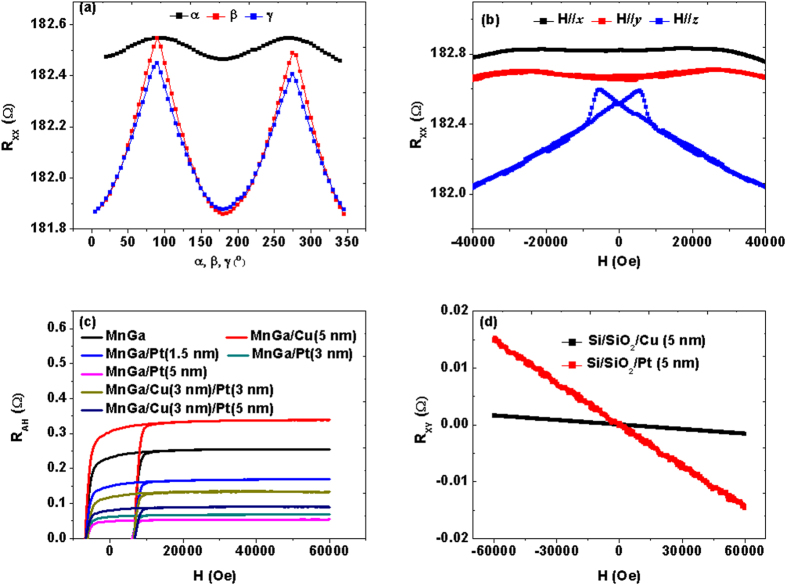
Magnetotransport properties of MnGa, MnGa/Cu and MnGa/Pt. (**a**) The α, β and γ dependence of R_XX_ for MnGa with a magnetic field of 6 T. (**b**) The field dependent R_XX_ with **H** along the ***x, y,*** and ***z*** directions respectively. (**c**) Anomalous Hall effect of MnGa, MnGa/Cu(5), MnGa/Pt(*t*) (*t* = 1.5 ~ 5 nm), MnGa/Cu(3)/Pt(3) and MnGa/Cu(3)/Pt(5). (**d**) Hall effect of the 5-nm-thick Pt and Cu layers grown on Si/SiO_2_ substrates.

Firstly, we measured the transport properties of a single MnGa layer. Figure 3a shows the α, β and γ dependence of R_XX_. R_XX_ (α) shows the sin^2^ (α) dependence while R_XX_ (β) and R_XX_ (γ) all adapt sin^4^ dependence on the angle. Figure 3b shows the field dependent resistance with **H** along the ***x, y**,* and ***z*** directions respectively. It is obviously seen from the two figures that the most dramatic resistance change happens when the magnetization is out of the plane, which is caused by the special domain structure of MnGa. Then we studied the thickness dependence of AHE resistance R_ΑΗ_ in Mn_1.5_Ga/Pt (*t*) (*t* = 1 ~ 5 nm), as compared with those of MnGa and MnGa/Cu shown in Fig. 3c. The R_ΑΗ_ was obtained by subtracting the ordinary Hall component (determined from a linear fit to the high-field region up to ±6 T). The Hall effect measurements of Pt (5 nm) and Cu (5 nm) grown on Si/SiO_2_ substrates show only the ordinary Hall effect with the Hall voltage linearly dependent on **H** as shown in Fig. 3d. The ordinary Hall effect is relatively small and will not dramatically influence the Hall effect in MnGa/Cu and MnGa/Pt. From Fig. 3c we can find that the *R*_ΑΗ_in MnGa/Cu (5) is larger than that in a single epitaxial MnGa film, while the *R*_ΑΗ_values in all the MnGa/Pt(*t*) bilayers become smaller. After inserting Cu between MnGa and Pt, the *R*_ΑΗ_ in MnGa/Cu(3)/Pt(3) and MnGa/Cu(3)/Pt(5) become larger than those in the films with direct contact but a little bit smaller than that in the single MnGa film. It has been proved that Cu is very far from the Stoner instability and the nonlocal exchange force does not reach over such a thickness. Meanwhile, Cu has a long (several hundred nanometers) spin diffusion length and a very small SHE due to weak SOC, and could carry spin current over a long distance. Altering the interface by inserting Cu can block the interfacial SOC induced by Pt. Therefore, the observation of the modified AHE cannot be explained by the influence of a magnetized Pt layer induced by magnetic proximity effect but mostly depend on the SHE of the heavy metals.

### Magnetotransport properties in MnGa/Pt/CoFe/Pt and MnGa/Cu/CoFe/Cu

**Figure 4:**
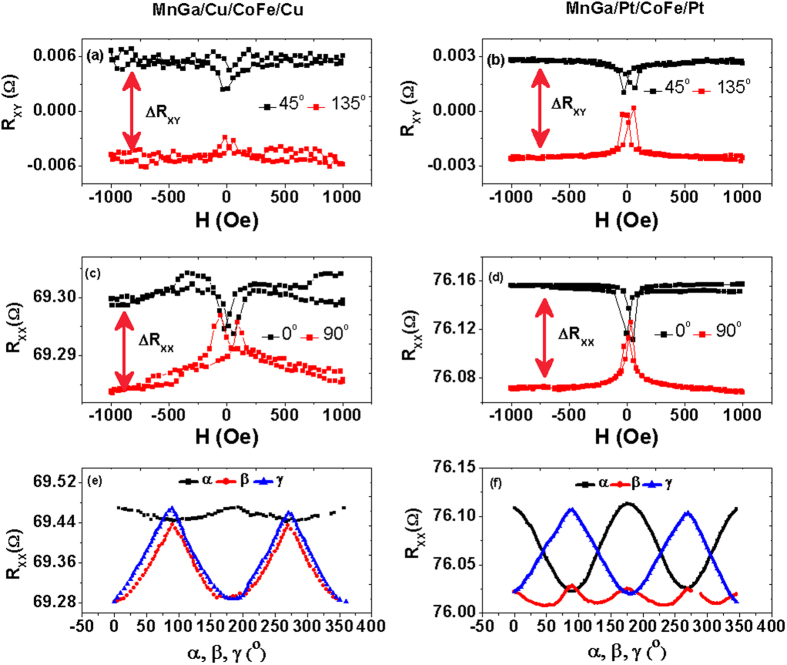
PHE and MR of MnGa/Cu/CoFe/Cu and MnGa/Pt/CoFe/Pt. (**a,b**) Measurements of R_XY_ in MnGa/Cu/CoFe/Cu and MnGa/Pt/CoFe/Pt with the applied magnetic field forming a fixed angle (α = 45° and α = 135°) with the current. (**c,d**) Measurements of R_XX_ with the applied magnetic field forming a fixed angle (α = 0° and α = 90°) with the current; (**e,f**) The 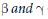 dependence of R_XX_ with a magnetic field of 6 T.

The most complicated transport properties have been observed in MnGa/Pt/CoFe/Pt, and the results of the multilayers and the reference sample MnGa/Cu/CoFe/Cu are shown in Fig. 4. Before carrying out the measurement of AMR and PHE at low field range, a high magnetic field of 6 T was firstly applied along ***z***axis of the samples to induce perpendicular magnetization of MnGa and then decreased to zero. As compared with Cu/CoFe/Cu, both ΔR_XX_ and ΔR_XY_ of MnGa/Cu/CoFe/Cu are very small as shown in Fig. 4a,c, which are also consistent with equation (1) and (2). However, the resistance changes in MnGa/Pt/CoFe/Pt become dramatic, especially for ΔR_XY._ The results are not consistent with the mechanism of either SMR or AMR. For the low field measurement, the strong and complex interfacial SOC have decreased PHE when Pt is sandwiched by the in-plane CoFe and PMA MnGa, but R_XY_ still shows the 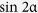 dependence. On the other hand, the angle-dependent MR of MnGa/Cu/CoFe/Cu measured at high field reveals a combination of Cu/CoFe/Cu and MnGa. R_XX_(α) shows the cos^2^(α) dependence while R_XX_ (β) and R_XX_ (γ) show similar angle dependence with those of MnGa, as shown in Fig. 4e. For MnGa/Pt/CoFe/Pt, R_XX_ (β) shows a distinctive behavior, which adapts the dependence of cos^2^ (2β). In this case, the magnetization is perpendicular to the current in the film plane all through the measurement, which indicates the combination of both complicated domain wall scattering and strong interfacial SOC when Pt is sandwiched between the in-plane magnetized CoFe and PMA MnGa films.

### High magnetic field dependent resistance

**Figure 5:**
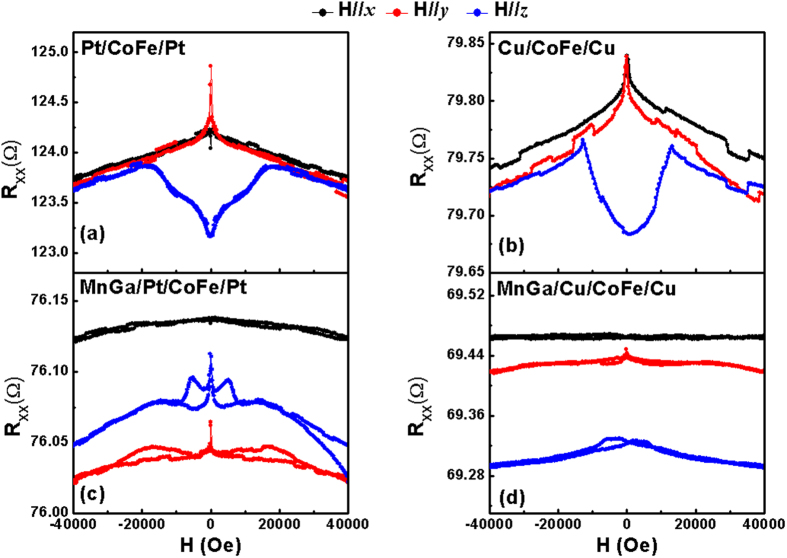
The high magnetic field dependent resistance R_XX_ of the four multilayers with a field H along the *x, y,* and *z* directions respectively.

**Figure 6:**
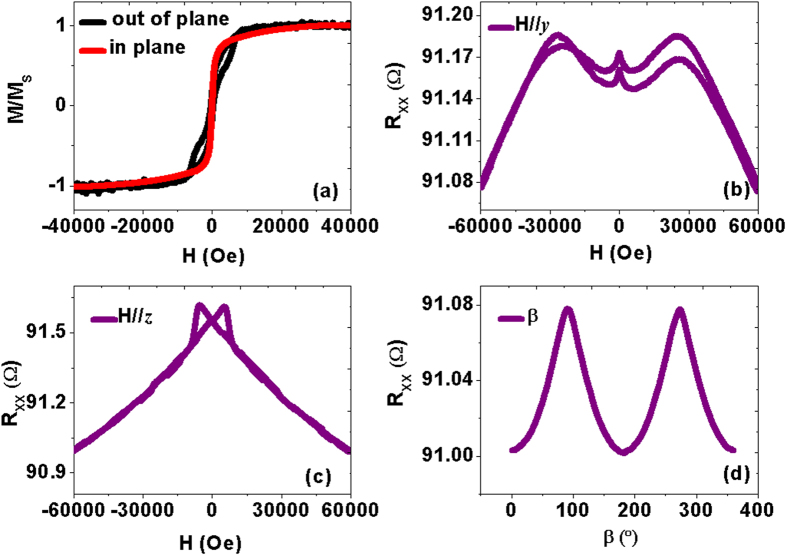
The magnetic and transport properties of MnGa/Pt(1.5)/CoFe/Pt(1.5). (**a**) The normalized out-of-plane and in-plane magnetic hysteresis loops; (**b,c**) The field dependent R_XX_ with **H** along the ***y***and ***z***directions respectively; (**d**) β dependence of R_XX_ with a magnetic field of 6 T.

To further study the transport properties induced by domain wall scattering, we also measured the high magnetic field dependent resistance of the four multilayers with a field **H** along the ***x, y,***and ***z*** directions respectively. In Fig. 5a,b, the in-plane curves (**H//*****x*** and **H//*****y***) of both Cu/CoFe/Cu and Pt/CoFe/Pt show steep resistivity changes at small fields (<1000 Oe), but for H//*z* the curves indicate coherent magnetization rotation which is completed at about 1.8 T. At large fields, the films become homogeneously magnetized, all the curves exhibit linear decrease which is usually referred to as the spin-disorder MR caused by the suppression of spin waves with increasing field strength^1^. It is indicated that the difference of domain wall scattering between Cu/CoFe/Cu and Pt/CoFe/Pt is not large. However, the field dependent resistance in MnGa/Pt/CoFe/Pt becomes much more complicated compared with that in MnGa/Cu/CoFe/Cu. When the current is applied along ***x*** directions, the high magnetic field dependence of R_XX_ for the two samples is almost the same. In contrast, when the magnetization is perpendicular to the current, for example **H//*****y*** and **H//*****z**,* more evident resistivity changes at small fields happen. To study the resistivity due to domain wall scattering, Levy and Zhang developed a quantum mechanical description based on the giant MR Hamiltonian which leads to an increased resistance due to the mixing of the spin conduction channels induced by magnetization rotation within the domain wall^23^. Noticeably, they were the first to derive both the CIW (current in wall) and CPW (current perpendicular to wall) resistances. Viret *et al*. carried out the low-temperature measurements of the resistance induced by magnetic domain walls in FePd with perpendicular anisotropy in the CPW and CIW configurations, which quantitatively agreed with the model of Levy and Zhang^24^. They have found that the resistance variation in these two configurations are quite different, which reflects the asymmetric domain wall induced increase of resistivity. Thus we ascribe the different resistance variations between current parallel (**H//*****x***) and perpendicular to the magnetic field (**H//*****y*** and **H//*****z***) to different domain wall rotation. Therefore, both strong SOC and domain wall scattering at the interfaces largely contribute to the transport properties of MnGa/Pt/CoFe/Pt and the bottom Pt layer sandwiched by MnGa and CoFe may play a dominant role. To further improve it, we investigate the magnetic and transport properties of MnGa/Pt(1.5)/CoFe/Pt(1.5) as shown in Fig. 6. Both out-of-plane and in-plane hysteresis loops reveal the existence of magnetic coupling but indicate different micromagnetic configurations of domain walls as compared with MnGa/Pt(5)/CoFe/Pt(1.5) shown in Fig. 1a. It has been found that the variations of the transport properties in the multilayers are not evident with ***H//x***, thus we just focus on the transport behavior when the current is perpendicular to the magnetic field. The complicated behaviors of the high magnetic field dependent resistance R_XX_ with **H//*****y*** also reveal the existence of complex domain wall scattering. However, for MnGa/Pt(1.5)/CoFe/Pt(1.5), the field dependent resistance R_XX_ shows similar behavior with that in the single MnGa film when ***H***//***z***, indicating weak contribution from magnetic coupling along the ***z***direction. Meanwhile, the β scan of R_XX_ also shows similar behavior with that in MnGa as shown in Fig. 3a. It is proved that decreasing the thickness of the bottom Pt layer decreases not only the contribution of magnetic coupling but also that from strong SOC. However, more detailed understanding of the transport properties in this kind of multilayer with different magnetic anisotropies is still a challenge and need further study.

### Current dependence of SMR

**Figure 7:**
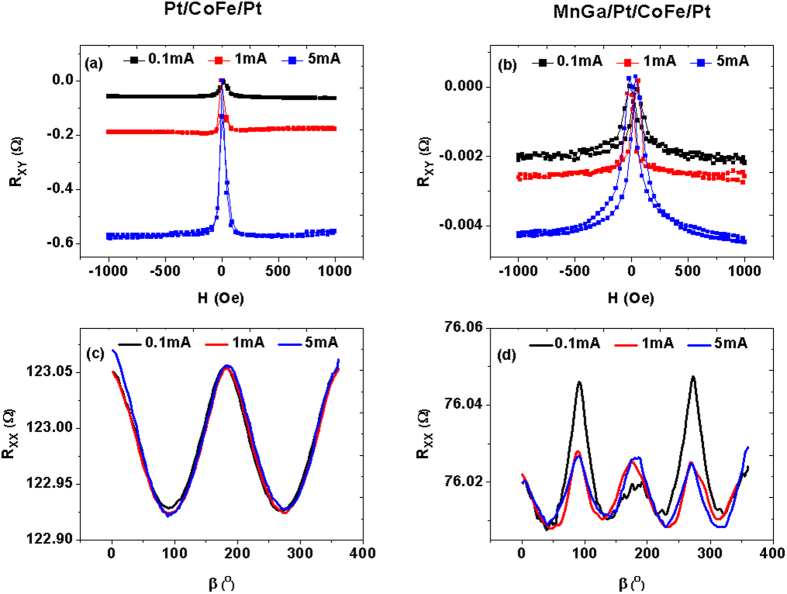
The current dependence of SMR. (**a,b**) Measurements of R_XY_ in Pt/CoFe/Pt and Pt/CoFe/Pt as α = 135° with the current of 0.1, 1 and 5 mA; (**c,d**) β dependence of R_XX_ with a magnetic field of 6 T and the current of 0.1, 1 and 5 mA for the two samples.

In our experiment, the current of 1 mA is applied and the current density is about 10^5^ A.cm^−2^. We have also carried out the current dependence of R_XX_ (β) and R_XY_ (H) in Pt/CoFe/Pt and MnGa/Pt/CoFe/Pt multilayers with the current of 0.1, 1 and 5 mA as shown in Fig. 7. The measurements of R_XY_ (H) were done with α =  135°. It is found that as increasing the current, the R_XY_ in both two samples are enhanced. However, the R_XX_ (β) with high magnetic field of 6 T are almost the same with different applied current for Pt/CoFe/Pt. For MnGa/Pt/CoFe/Pt, when the current is 0.1 mA, the R_XX_(β) reveals a more evident contribution from MnGa film as shown in Fig. 3a, while R_XX_(β) are also almost the same with 1 and 5 mA. It is indicated that the SHE may be not the sole origin of the SMR effect, other contributions of NM/FM interfaces, such as, texture induced geometrical size^10^ or interfacial Rashba effect^25^,^26^ may be existent, and further study is required to clarify the origin.

In summary, we have investigated the origin of the hybrid MR in Pt/CoFe/Pt, MnGa/Pt and MnGa/Pt/CoFe/Pt multilayers. Both the PHE measured at low field and the angle-dependent MR at high field in Pt/CoFe/Pt revealed the combination of SMR and normal AMR, indicating the large contribution of strong SOC at the interfaces. For MnGa/Pt, the strong interfacial SOC between Pt and PMA MnGa decreased the effective AHE. The MR in MnGa/Pt/CoFe/Pt was not a simple combination of SMR and AMR, but ascribed to the complicated domain wall scattering and strong SOC when Pt was sandwiched between the in-plane magnetized CoFe and PMA MnGa films. Our results provide a way of modulating the spin-related transport effect when strong SOC metals contact with different magnetic anisotropy metals.

## Methods

In the experiment, Pt (5)/CoFe (10)/Pt (1.5) (in nanometer) and a reference sample Cu (5)/CoFe (10)/Cu (1.5) were deposited on Si/SiO_2_ substrates by dc magnetron sputtering. 20-nm-thick MnGa films were grown on GaAs(001) substrates by molecular beam epitaxy^21^, then Pt(*t*) (*t* = 1.5 ~ 5), Cu(5), Pt(5)/CoFe(10)/Pt(1.5), Pt(1.5)/CoFe(10)/Pt(1.5) and Cu(5)/CoFe(10)/Cu(1.5) were grown on MnGa by dc magnetron sputtering, respectively. The magnetic and transport properties were carried out at room temperature using a superconducting quantum interference device and a physical property measurement system respectively.

## Additional Information

**How to cite this article**: Meng, K. *et al*. Hybrid magnetoresistance in Pt-based multilayers: Effect originated from strong interfacial spin-orbit coupling. *Sci. Rep.* **6**, 20522; doi: 10.1038/srep20522 (2016).
